# The Story of the Insane from Year to Year

**Published:** 1903-09-26

**Authors:** 


### THE STORY OF THE INSANE FROM YEAR TO YEAR.

Sixteenth Annual Series.

### THE CITY OF LONDON ASYLUM.

This asylum contained 512 patients on the first of January, and by the end of the year the number had risen to 542. There were 185 first admissions, 13 re-admissions, 47 discharged recovered, 59 discharged relieved, and 55 died. The average number resident was 518, and the total number under treatment was 707. The recoveries were 30·72 per cent. of the admissions, and the deaths 10·61 per cent. of the average number resident. The first of these percentages is lower than is usual in county asylums, and the second is a little higher. Of the year's deaths 15 were caused by cerebral and spinal diseases, only 1 by pneumonia, 6 by consumption, 4 by dysentery, and 1 by enteritis. Of the admissions various moral causes account for the insanity in 33 instances, and among the physical causes hereditary influence heads the list with 60, previous attacks come next with 49, and drink with 21. The low recovery rate is explained by the facts that many of the admissions had been treated elsewhere before being sent to the City of London, and that 100 of the admissions were either chronic patients or cases in which previous attacks of insanity had existed. This is one of our public asylums which admits a large number of private patients. There were 207 of this class in residence at the end of the year. In the year 1892 the number was only 18, and it has kept steadily rising year by year until the present time; and it seems likely that it will continue to do so until the limits of accommodation for such cases are reached. During the small-pox epidemic Dr. White took every possible precaution to prevent the entry of the disease into his asylum, and it is satisfactory to learn that he was successful in keeping it at bay. There were, however, 3 sporadic cases of enteric fever; 1 of these was a nurse, and the others were patients. The nurse and 1 patient died. Every effort was made to trace the source of infection, but no cause could be found. That asylum plague, dysentery or colitis, made its appearance in 6 patients, of whom 4 died. The lectures on mental nursing continue to be delivered by Dr. Patterson; and 5 of the staff obtained the certificate of the Medico-Psychological Association. It does not appear that Dr. White has as yet inaugurated a three years' course of instruction to his staff, which we more especially regret as he was one of the first medical superintendents in England to systematically train his attendants and nurses for their duties. The head attendant resigned during the year and received a pension of £40. The Visitors record their "appreciation of the continued loyal and efficient manner in which the medical superintendent discharges the onerous duties of his office." The weekly maintenance charges are—for home patients, 12s. 3d.; for out-county, 14s.; and for private cases, from 21s. to 42s.

### THE DERBY BOROUGH ASYLUM.

Here the numbers were 319 on the first of January, and 325 on the 31st of December. There were 87 first admissions, 9 re-admissions, 42 recoveries, 11 discharged relieved, and 27 died. The average daily number resident was 324, and the total number under treatment was 413. The recoveries work out at the high percentage of 45·1 on the year's admissions, and the deaths at 8·3 on the average number resident. Of the year's deaths, 8 were caused by cerebral and spinal diseases, 1 by pneumonia, and 4 by consumption. Of the admissions, Table X. shows that in 34 cases the insanity was owing to moral causes, in 28 to hereditary influence, and in 24 to intemperance in drink. The patients admitted to our county and borough asylums are often in very feeble health, and those admitted to this asylum were worse even than the average. Two-thirds were suffering from recognisable physical disease, and only 10 were in good health. Dr. Macphail mentions that "25 of the patients who recovered were under treatment from one to six months; 12 from six months to a year, and only 5 recoveries were obtained after over a year's residence. The importance of early treatment in insanity is thus emphasised." In English public asylums the death-rate from consumption is about 15 per cent. of the total deaths; and in the Derby Asylum it has only been 9·4 per cent. since its opening fourteen years ago. The asylum is full, and even the Isolation Hospital has been used for convalescent cases; but a new block is being built for private patients which, when opened, will reduce the overcrowding; and, if need be, the out-county cases could be discharged. There are only 23 of these, so that the relief in any case would be of a very temporary nature. The annual increment of the insane in asylums is about 3 per cent. on the total number of patients. Derby borough has therefore to provide for about 9 additional cases every year; and it is quite time that additions were being put up, or the inevitable "boarding-out" will soon have to be tried to put off the evil day of bricks and mortar. Dr. Macphail was the first English medical superintendent to try the Brabazon Employment Scheme for his patients, and he speaks highly of it. "It has had the effect of brightening the lives of many of the patients, and has helped in the cure of not a few." The Visitors say that "All articles of consumption, and also material for wearing apparel, bedding, etc., as far as possible, are purchased from tradesmen in the town." Unless the Derby Committee be exceptionally fortunate, it is certain that this is a most faulty system, and is one which usually means the enrichment of a few tradesmen at the expense of the bulk of the ratepayers of the district; whereas it is not the few, but the many, that every board of management should study, even at the risk of a little temporary unpopularity. The Visitors further say they have pleasure "in renewing the expression of their entire satisfaction with the manner in which Dr. Macphail continues to discharge the responsible and onerous duties which devolve upon him," and in this expression of opinion everyone will agree with them. The weekly charge for maintenance is 11s. 1d. per head for borough patients, and for out-borough and private patients from 14s. to 17s. 6d.

It has been decided to erect a new Presbyterian church in Pretoria as a memorial to the soldiers, volunteers, doctors, and nurses of that communion who died while in service in South Africa.

